# Toxicity of *Evodiae* *fructus* on Rat Liver Mitochondria: The Role of Oxidative Stress and Mitochondrial Permeability Transition

**DOI:** 10.3390/molecules191221168

**Published:** 2014-12-16

**Authors:** Qingyan Cai, Jingjing Wei, Wei Zhao, Si Shi, Yu Zhang, Renrong Wei, Yue Zhang, Weirong Li, Qi Wang

**Affiliations:** Institute of Clinical Pharmacology, Guangzhou University of Chinese Medicine, Guangzhou 510405, China; E-Mails: cqywho@163.com (Q.C.); weijingjingfhl@163.com (J.W.); athenaty@gzucm.edu.cn (W.Z.); m18825141524@163.com (S.S.); zy970838172@163.com (Y.Z.); weirenrong@126.com (R.W.); yueyazy@hotmail.com (Y.Z.)

**Keywords:** *Evodiaefructus* (EF), hepatotoxicity mechanisms, oxidative stress, mitochondrial permeability transition (MPT), HPLC/UV

## Abstract

*Evodiae* *fructus* (EF) has been used in China for thousands of years as an analgesic, antiemetic, anti-inflammatory and antidiarrheal drug. EF is a toxic drug and causes hepatotoxicity in humans. Although recent chronic toxicity studies performed on aqueous extract of EF has revealed that it can produce obvious cumulative hepatotoxicity, the mechanism behind this toxicity is still uncertain. In the present study, we investigated the influence of EF on oxidative stress, mitochondrial permeability transition, adenosine triphosphate (ATP), and cytochrome C release of hepatic mitochondria. Rats were divided into four groups and fed distilled water, 6, 12, 24 g/kg of aqueous extract of EF daily for 15 days. Evodiamine, rutaecarpine and evodine were quantified in the aqueous extract by high performance liquid chromatography with ultraviolet detection (HPLC/UV). The results showed that aqueous extract of EF could significantly (*p* < 0.05) decrease MnSOD levels to 56.50%, 46.77% and 19.67% of control group, GSH level was decreased to 74.24%, 53.97% and 47.91% of control group and MDA level was increased to 131.55%, 134.34% and 150.81% of control group in the 6, 12 and 24 g/kg groups, respectively; extract also induced mitochondria swelling, vacuolation, MPT pore opening and a significant decrease (*p* < 0.05) in mitochondrial potential, while ATP levels were significant decreased (*p* < 0.05) to 65.24%, 38.08% and 34.59% of control group in the 6, 12 and 24 g/kg groups, respectively, resulting in ATP depletion and CytC release, finally trigger cell death signaling, which are the partial hepatotoxicity mechanisms of EF.

## 1. Introduction

*Evodiae* *fructus* (EF, known in Chinese as Wu Zhu Yu), is the dried, nearly ripe fruit of *Evodia* *rutaecarpa* (Juss.) Benth. The plant is a herb used in Traditional Chinese Medicine for gastrointestinal disorders, cardiovascular diseases, headache, amenorrhea, abdominal pain, dysentery, and postpartum hemorrhage [1,2]. EF has been used for thousands of years in China, mainly used in prescriptions; its therapeutic efficacy is supported by clinical patients but lacks evidence-based medicine (EBM) proof, which are encouraging for both prescription and individual traditional Chinese medicine (TCM) herbs. Alkaloids are major bioactive ingredients in EF, and many recent studies have focused on the pharmaceutical potential of these alkaloids, such as the major alkaloid evodiamine, which induces apoptosis and G2/M arrest in human colorectal carcinoma cells [3], inhibits the growth of human colon carcinoma cells [4] and hepatoblastoma cell lines [5]. Rutaecarpine, another alkaloid in EF, also exhibits interesting pharmaceutical activity, such as suppressing atherosclerosis [6], and vasodilation for treatment of cardiovascular disorders [7]. Another study investigated the antioxidant activity of the total alkaloids in EF [8].

However, in China’s most ancient herbal medicine book “Shen Nong’s Herbal Classic” the mild toxicity of EF has been noted. EF can cause toxic symptoms such as stomach ache, vomiting, blurred vision, *etc.* by overdose of EF in prescriptions in a case of chronic esophagitis [9] and causes hepatotoxicity in humans [10,11,12]. Recent experimental studies have confirmed that long-term use of high doses of EF aqueous extract will produce obvious cumulative toxicity and find that the toxicity mainly affects the liver [13,14]. The levels of some biochemical indicators of liver function change and the liver weight and the ratio of liver to body weight change, and hepatic tissue damage was obvious in histopathologic examinations of hepatic tissue in rats [15]. Multiple intragastric administrations of water-extracted components of EF at certain dosages may induce acute hepatotoxical injury in mice and show a certain dosage-time-toxicity relationship [16]. Certainly there are different reports that state that no adverse effects are observed in rats after evodia fruit powder administration, and the discrepancy was attributed to dose levels, administration period and test substance and species [17]. However, there are no reports describing the toxicological mechanisms of EF.

Drug-induced hepatotoxicity ranges from asymptomatic, mild, nonspecific biochemical changes to acute hepatitis, chronic hepatitis, acute liver failure, cholestasis, liver cirrhosis, and even liver cancer [16]. In general, the mechanisms of drug-induced liver injury are divided into liver damage and idiosyncratic reactions. Some drugs or their metabolites damage liver cells directly or act on the transporters of the bile duct, which can lead to bile duct obstruction and cholestasis, while other drugs can cause immune-mediated liver damage and lead to inflammation and liver damage [18,19,20,21], and other drugs trigger mitochondrial dysfunction that evolves into liver cell apoptosis or necrosis [22,23]. Mitochondrial dysfunction is a major mechanism of liver injury induced by drugs. Mitochondrial permeability transition (MPT) plays a pathogenetic role in the mitochondria-mediated hepatocyte injury. MPT is characterized by a progressive permeabilization of the inner mitochondrial membrane dependent on the excessive amount of intramitochondrial Ca^2+^ and results in mitochondrial swelling, decrease in mitochondrial Δ*Ψ* and release of accumulated Ca^2+^, outer mitochondrial membrane rupture and release of cytochrome C (CytC) [24,25].

In the present study, we investigated the capability of EF to induce oxidative stress and MPT by using mitochondria isolated from the liver of rats which were orally administered aqueous extract of EF for 15 days. Our results may help understand the relationship between hepatotoxicity and the oxidative stress linked with mitochondrial dysfunction. Evodiamine, rutaecarpine and evodine, the major components of EF, were quantified by high performance liquid chromatography with ultraviolet detector (HPLC/UV) analysis of EF.

## 2. Results

### 2.1. Quantification of Evodiamine, Rutaecarpine and Evodine by HPLC/UV

**Figure 1:**
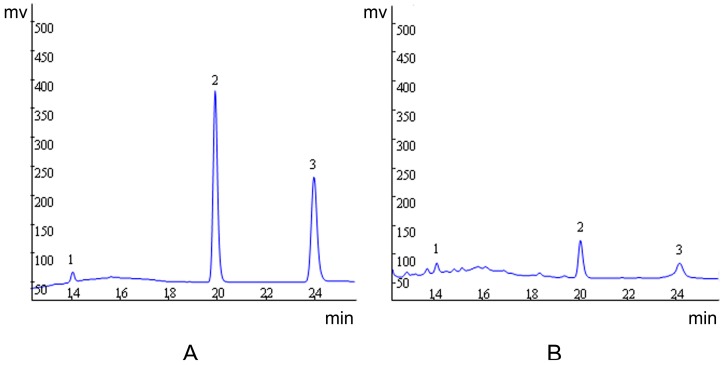
Representative high performance liquid chromatography profile of standard (**A**) and aqueous extract of EF (**B**). Evodine (1), evodiamine (2) and rutaecarpine (3).

Evodiamine, rutaecarpine and evodine were quantified. The results revealed a higher concentration of evodine in comparison to the other compounds. The concentration for each substance is given in Table 1. We also show the corresponding chromatogram in Figure 1.

**Table 1:** The contents of evodiamine, rutaecarpine and evodine in EF

Ingredient	Extract	
	mg/g	%
Evodiamine	0.096 ± 0.011	0.009 ± 0.001
Rutaecarpine	0.044 ± 0.001	0.004 ± 0.001
Evodine	7.473 ± 0.241	0.747 ± 0.024

Data are presented as mean ± SEM (n = 3).

### 2.2. Effect of EF on Manganese Superoxide Dismutase (MnSOD), Glutathione (GSH), and Malondialdehyde (MDA)

**Figure 2:**
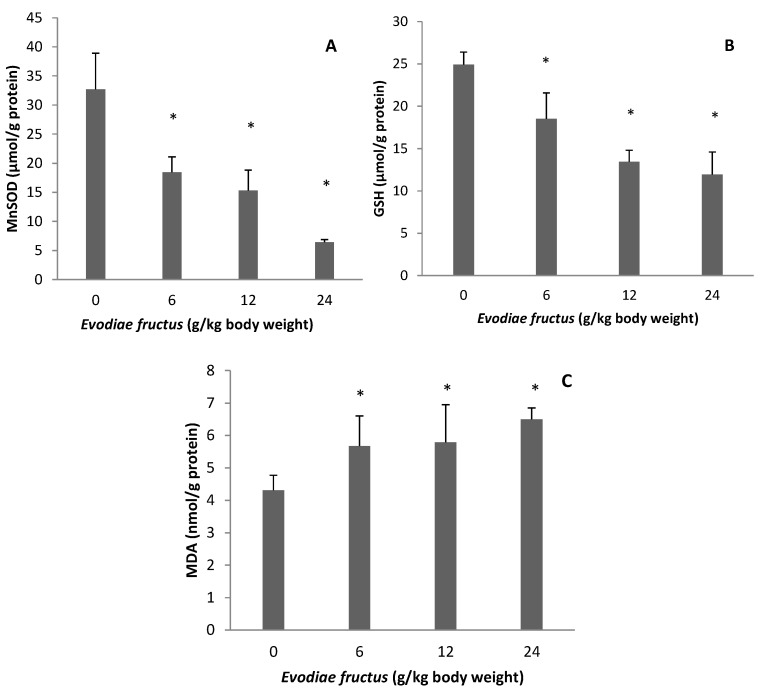
Effects of aqueous extract of EF on mitochondrial MnSOD (**A**), GSH (**B**) and MDA (**C**). Data were presented as mean ± SEM (n = 10). Compared with control group * *p* < 0.05.

As shown in Figure 2, MnSOD level was decreased to 18.47 ± 2.62, 15.29 ± 3.53 and 6.43 ± 0.46 µmol/g protein, GSH level was decreased to 18.50 ± 3.07, 13.45 ± 1.36 and 11.94 ± 2.67 µmol/g protein and MDA level was increased to 5.67 ± 0.93, 5.79 ± 1.16 and 6.50 ± 0.35 nmol/g protein in the samples from rats treated with 6, 12 and 24 g/kg body weight, respectively, whereas in the control group, the level was 32.69 ± 6.23, 24.92 ± 1.48 and 4.31 ± 0.46 µmol/g protein, respectively. Therefore MnSOD level was decreased to 56.50%, 46.77% and 19.67% of control group, GSH level was decreased to 74.24%, 53.97% and 47.91% of control group and MDA level was increased to 131.55%, 134.34% and 150.81% of control group in the 6, 12 and 24 g/kg groups, respectively.

### 2.3. Effect on Mitochondrial Permeability Transition

**Figure 3:**
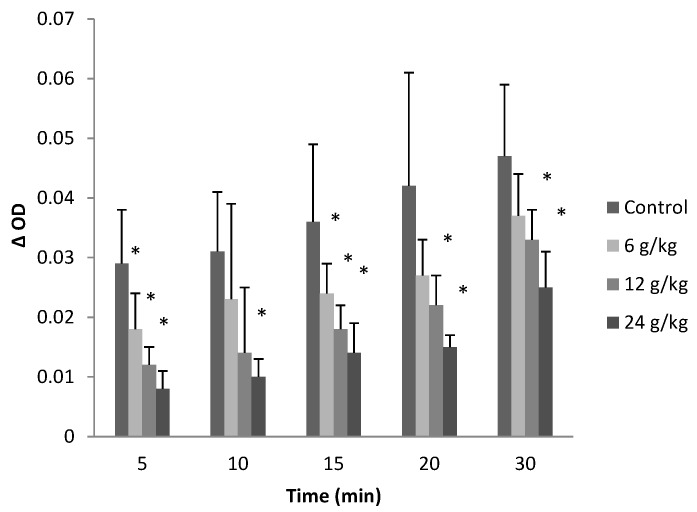
Effects of aqueous extract of EF on mitochondrial permeability transition. Figure showed the decrease in absorbance at 540 nm at different time points of the mitochondria which induced by 20 µmol/L CaCl_2_. Data were presented as mean ± SEM (n = 10). Compared with control group * *p* < 0.05.

Incubation of energized mitochondria which were treated by aqueous extract of EF with succinate in the presence of Ca^2+^ (20 µmol/L) induced a larger-amplitude swelling than in the control group, and dose-dependent mitochondrial swelling was observed at different time points after incubation, ΔOD value reduced to some extent (*p* < 0.05) as shown in Figure 3. The absorbance decreased by 24 g/kg of EF to 27.59%, 32.36%, 38.89%, 35.71% and 53.19% of control group at 5, 10, 15, 20 and 30 min, respectively.

### 2.4. Electron Microscopy Findings

**Figure 4:**
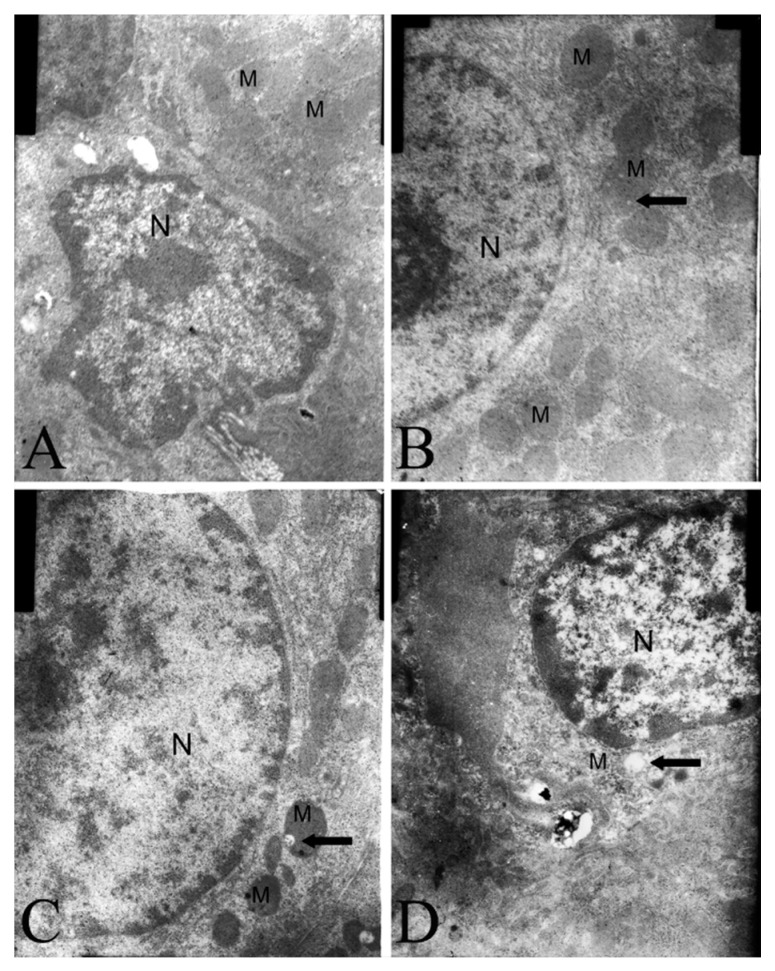
Mitochondrial changes in the rat liver treated with control rat (**A**), aqueous extract of EF 6 g/kg/day (**B**), 12 g/kg/day (**C**) and 24 g/kg/day (**D**) last 15 days. M mitochondria, N nucleus, the arrows showed vacuolation. Magnification 15,000×.

In the liver cells there were lesions in the mitochondria of aqueous extract of EF-treated rats compared to distilled water-treated animals. The effect of aqueous extract of EF was apparent in the vacuolated mitochondria (Figure 4).

### 2.5. Effect on Mitochondrial Transmembrane Potential

When the mitochondrial membrane potential is high, JC-1 gathered in the mitochondrial matrix and formed J-aggregates which can produce red fluorescence; when the mitochondrial membrane potential is low, JC-1 cannot be gathered in the mitochondrial matrix and existed as the monomer which can produce green fluorescence.

**Figure 5:**
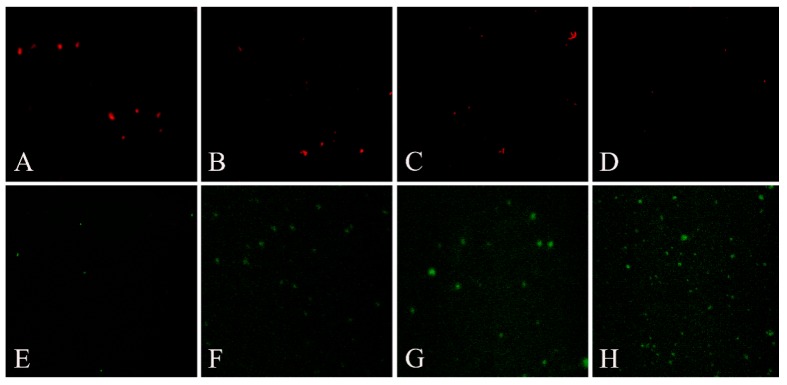
Effects of aqueous extract of EF on mitochondrial transmembrane potential by fluorescence microscope. The picture **A**–**D** are red fluorescence of groups of control, 6, 12, 24 g/kg aqueous extract of EF treated rat, successively; the **E**–**H** are green fluorescence correspond to the red.

According to the results of fluorescence microscopy, the green fluorescent area significantly increased and red fluorescence decreased in the groups of aqueous extract of EF, these results as shown in Figure 5A–H indicated that mitochondrial membrane potential decreased significantly in the groups of aqueous extract of EF. 

**Figure 6:**
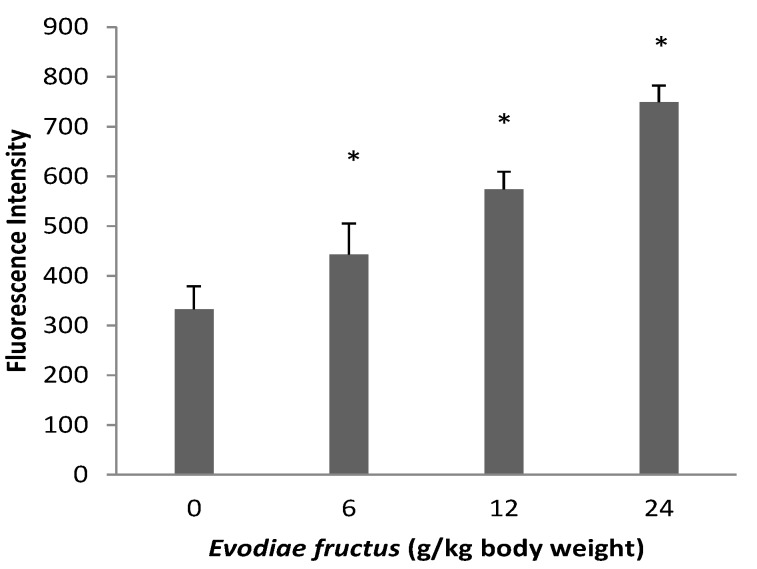
Effects of aqueous extract of EF on mitochondrial transmembrane potential by fluorescence spectrophotometer. The changes of fluorescence intensity of different groups were showed. Data were presented as mean ± SEM (n = 10). Compared with control group * *p* < 0.05.

Fluorescence spectrophotometer detection results are shown in Figure 6. The fluorescence intensity of JC-1 monomer was monitored at the 490/530 nm wavelength pair. The fluorescence intensity of aqueous extract of EF groups was obviously higher in a dose dependent manner than that of control group, which means that the JC-1 monomer level in the aqueous extract of EF groups was higher, indicating that aqueous extract of EF caused a decrease in mitochondrial membrane potential.

### 2.6. Effect of EF on ATP

**Figure 7:**
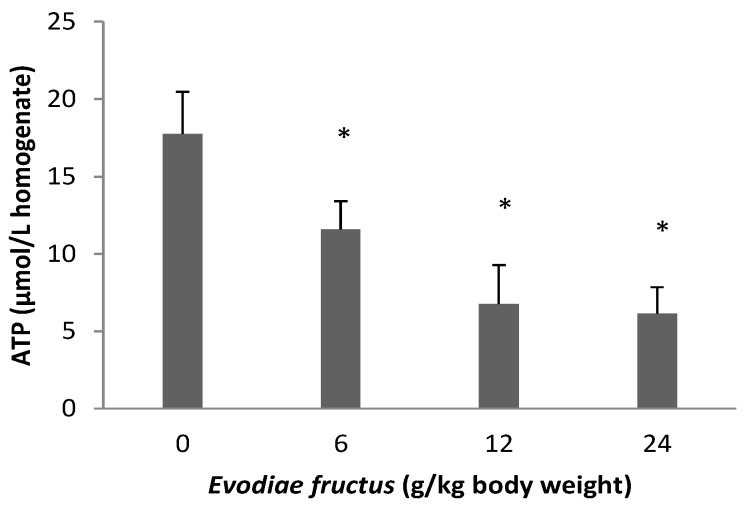
Effects of aqueous extract of EF on liver tissue ATP. Data were presented as mean ± SEM (n = 10). Compared with control group * *p* < 0.05.

As shown in Figure 7, the ATP level was decreased to 65.24%, 38.08% and 34.59% of control group, which values were 11.58 ± 1.83, 6.76 ± 2.52 and 6.14 ± 1.69 µmol/L liver tissue homogenate in the samples treated with 6, 12 and 24 g/kg body weight, respectively, whereas in the control group, the level was 17.75 ± 2.72 µmol/L liver tissue homogenate. 

**Figure 8:**
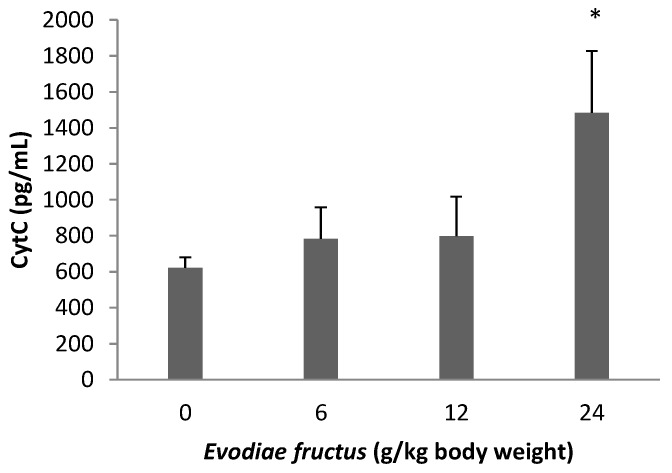
Effects of aqueous extract of EF on CytC release. The concentrations of CytC in endochylema of different groups were showed. Data were presented as mean ± SEM (n = 10). Compared with control group * *p* < 0.05.

### 2.7. Effect on CytC Release

Concentrations of CytC in endochylema of the mitochondria which were isolated from the liver of rats are shown in Figure 8. The concentration of CytC in the aqueous extract of the EF group with 24 g/kg was obviously higher than in the control group, the other groups of aqueous extract of EF showed no obvious change compared with the control group.

## 3. Discussion

Although hepatotoxicity caused by herbal TCMs has not received serious attention, more cases have been published in recent years, providing new clinical challenges. In the past three years, new cases of hepatotoxicity caused by herbal TCM were reported from nearly 20 countries, focusing on nine TCM herbal mixtures and four individual TCM herbs with potential health hazards. Among them was the herbal TCM Wu Zhu Yu [26]. EF is a widely used herb medicine that has already been demonstrated to present hepatotoxicity in humans that is also reproducible in rats, and 35 days of EF use will produce obvious hepatotoxicity, presenting elevated alanine aminotransferase and aspartate aminotransferase levels, along with pathological changes in liver tissue [15]. However there is little knowledge about the mechanism of this toxicological side-effect. Hepatotoxicity is a serious problem during drug development and for the use of many established drugs. Although drugs can cause hepatotoxicity in different ways, mitochondrial dysfunction is one major mechanisms underlying drug-induced liver injury. Drugs or their reactive metabolites can trigger a mitochondrial oxidant stress, which leads to the opening of mitochondrial MPT pores. MPT can cause severe ATP depletion and necrosis, or lead to cytochrome c release, caspase activation, and apoptosis [27,28].

Alkaloids are the major bioactive compounds in EF, and pharmacological and clinical studies have indicated that the alkaloids from EF had diverse bioactivities. Evodiamine, rutaecarpine and evodine are specified as the biomarkers for quality assessment of EF in the Chinese Pharmacopoeia. These three compounds may also be involved in EF toxicity, as evodiamine could induce apoptosis and show cytotoxic effects [29], rutaecarpine in high doses may cause hepatic and renal toxicity on human normal hepatic cells and human embryonic kidney cells [30], evodiamine, rutaecarpine and evodine may cause nephrocyte toxicity in human embryonic kidney 293 cells, while synephrine could not [31]. Therefore we quantified levels of evodiamine, rutaecarpine and evodine by HPLC/UV, and the results showed that evodine was present in higher levels than evodiamine and rutaecarpine. Further research is needed on the relationship between ingredients and toxicity.

Oxidative stress is one of the most commonly invoked cell death mechanisms in all organ injury, including drug-induced liver damage. When considering reactive oxygen species-induced liver injury in any pathogenesis including drug-induced toxicity, lipid peroxidation (LPO) is the most invoked mechanism of cell death [32]. Therefore, cells have developed a sophisticated, multilayered defense system that is vital for cell survival in an oxygen environment. Superoxide can be removed by catalyzing the transfer of an electron from one superoxide molecule to another by Cu/Zn-superoxide dismutase, which is located in the cytosol, or by MnSOD present in mitochondria. Hydrogen peroxide can be reduced by glutathione peroxidases in the cytosol and mitochondria using electrons from GSH [33], while depletion of GSH decreases the antioxidant capacity and leads to oxidative stress [34]. LPO generates a wide variety of end products, including MDA, which is used as a marker of oxidative damage. The results obtained show clearly that EF increased MDA contents and deceased antioxidant enzymes MnSOD and GSH, reflecting hepatic mitochondrial oxidative damage (Figure 2).

The most critical effect of oxidant stress is the opening of the mitochondrial MPT pores, leading to the collapse of membrane potential and cessation of ATP synthesis [35]. The MPT pores are located at contact sites between the outer and inner mitochondrial membranes, and the mitochondrial permeability transition pore opening causes MPT [36]. Several drugs can trigger MPT in liver mitochondria, leading to apoptosis and/or necrosis. These drugs cause ‘cytolytic’ hepatitis, leading occasionally to fulminating hepatic failure. Some drugs (or metabolites) can directly trigger MPT in isolated mitochondria [37,38]. Other drugs such as tolcapone lead to liver cells with apparent swollen mitochondria, deformed or broken cristae and reduced matrix density [39]. The flow of electrons within the mitochondrial respiratory chain is coupled with the extrusion of protons from the mitochondrial matrix to the intermembrane space, which creates the mitochondrial transmembrane potential, ΔΨ. The membrane potential across the mitochondrial inner membrane is the driving force for phosphorylation of ADP in the process of oxidative phosphorylation [40]. Decrease of mitochondrial transmembrane potential is an irreversible cause of early apoptosis [41]. In the present study, incubation of energized mitochondria with EF in the presence of Ca^2+^ resulted in mitochondria swelling, vacuolation, MPT pore opening (Figure 3 and Figure 4), and mitochondrial mitochondrial ΔΨ decrease (Figure 5 and Figure 6), demonstrating that EF induces MPT.

Decreases in the electric potential difference of the exterior and interior mitochondrial membrane can result in a series of biochemistry changes between the exterior and interior mitochondria membrane, such as cessation of ATP synthesis, induction of CytC release, activation of the caspase family, release of apoptosis-inducing factors, which also results in an apoptotic cascade reaction and further results in cell apoptosis. For instance, troglitazone and acetaminophen activate c-Jun N-terminal protein kinase, thus inducing the cleavage of Bid, the translocation of this pro-apoptotic protein to the outer mitochondrial membranes and the release of CytC from mitochondria [42,43]. Our results showed that the exposure of liver mitochondria to EF results in an abrupt depletion of ATP (Figure 7), furthermore the high dose group with 24 g/kg could induce CytC release (Figure 8), following impairment of mitochondrial function related to alterations of the mitochondrial ΔΨ and oxidative phosphorylation with widespread LPO occurring at an early stage. 

## 4. Experimental

### 4.1. Plant Material and Extract Preparation

EF was obtained from the Kangmei Pharmaceutical Limited Company, Guangzhou, China. EF originated in Sichuan Province of China (lot number 110506851) and was authenticated by Professor Chenchen Zhu at Guangzhou University of Chinese Medicine as nearly ripe fruit of *Evodia* *rutaecarpa* (Juss.) Benth. Voucher specimens are preserved in Institute of Clinical Pharmacology, Guangzhou University of Chinese Medicine, Guangzhou, China. The powdered EF was soaked with water for 1 h before decocting two times with 10× water for 2 h at about 80°C. The filtrate which had filtered with gauze was combined and concentrated to 2.4 g crude drug per milliliter and stored at 4 °C. The concentrated solution was diluted to the required concentration (0.6, 1.2, 2.4 g crude drug/mL, respectively) with distilled water before use.

### 4.2. Chemicals

All chemicals were of analytical grade. Evodiamine (lot number w-012-120418) was purchased from Chengdu Ruifensi Biotechnology Limited Company, Chengdu, China. Rutaecarpine and evodine were purchased from the National Institute for the Control of Pharmaceutical and Biological Products, Beijing, China (lot numbers 110801-201006, 110800-20040).

### 4.3. HPLC Analysis

Evodiamine, rutaecarpine and evodine are the main active ingredients in EF. The contents of evodiamine, rutaecarpine and evodine in aqueous extract were determined by HPLC on an apparatus consisting of a Waters 515 pump and Waters 486 UV detector (Waters Corporation, Milford, MA, USA). Chromatography conditions: Synergi Hydro-RP C_18_ column (Phenomenex) (4.6 mm × 250 mm; 4 µm); Mobile phase: (A) methanol; (B) water; gradient elution (Time, A%): (0 min, 40%; 12 min, 75%; 26 min, 75%; 30 min, 40%; 37 min, 40%) and flow rate was 1.0 mL/min; Column temperature: 30 °C; UV detection: 230 nm; Injection volume: 20 μL. Calibration curve for evodiamine: *Y* = 125639 *X* − 50313 (*r* = 0.9998); rutaecarpine: *Y* = 92911 *X* − 52811 (*r* = 0.9997) and evodine: *Y* = 1326 *X* − 74814 (*r* = 0.9996).

### 4.4. Animals

Adult male rats of Sprague Dawley strain weighing 150 to 180 g were obtained from the Guangdong Medical Experimental Animal Center, Guangzhou, China. Animals were housed five per cage in a room maintained at 22 ± 0.5 °C with an alternating 12 h light-dark cycle. The animals were allowed free access to food pellets and water and were acclimatized for 1 week prior to usage. Experiments were performed during the light phase of the cycle (10:00–17:00). The animal protocols used in this work were evaluated and approved by the Animal Use and Ethic Committee of Guangzhou University of Chinese Medicine. They are in accordance with guidelines of the National Law for Laboratory Animal Experimentation.

### 4.5. Experimental Design

Animals were randomly divided into four groups, each containing 10 animals. Group 1 served as the control and received distilled water by oral gavage daily for 15 days. Group 2–4 received aqueous extract of EF as described previously for 15 days. Three dose levels were used (6, 12, 24 g/kg body weight). The dose selection criteria for EF are based on the preliminary experiment of chronic toxicity for aqueous extract of EF.

### 4.6. Isolation of Liver Mitochondria

Liver mitochondrial fraction was prepared according to the method described by the specification of mitochondria of rat liver tissue extraction kit (BIOBOX Bioscience Technology Limited Company, Nanjing, China). The livers (100–200 g) were isolated and cut to small cubes with scissors in 1.5 mL of the lysis buffer and homogenized twenty times in a Potter homogenizer. The homogenates were centrifuged at 800× *g* for 5 min kept at 4 °C. The resulting supernatant was decanted into a cold centrifuge tube to which solution A (0.5 mL) was added beforehand and further centrifuged at 15,000× *g* for 10 min. The resulting supernatant is endochylema and was transferred to another centrifuge tube. The pellet is mitochondria and was suspended in 0.1 mL of stock solution. Janus Green B (Sigma, St. Louis, MO, USA) was used to assess whether the mitochondria can be extracted successfully. The whole process was completed on ice; the lysis buffer, solution A and stock solutions were all part of the kit.

### 4.7. Assessment of MnSOD, GSH, MDA and ATP

MnSOD activity, GSH, MDA and ATP contents were determined with commercial kits (Jiancheng Bioengineering Institute, Nanjing, China) using a multi scan spectrophotometer to detect the absorbance and the concentration was calculated according to the corresponding standard curve.

### 4.8. Assessment of Mitochondrial Permeability Transition

Reaction medium (pH 7.2) containing liver mitochondrial protein (0.5 mg/mL), sucrose (125 mmol/L), KCl (50 mmol/L), HEPES (5 mmol/L), KH_2_PO_4_ (2 mmol/L), MgCl_2_ (1 mmol/L) was preincubated at 30 °C in the presence of CaCl_2_ (20 µmol/L). The mitochondria were energized with 5 mmol/L succinate. Mitochondrial swelling as the indicator of MPT was estimated from the decrease in absorbance at 540 nm [40].

### 4.9. Electron Microscopy

Samples for transmission electron microscopy were taken from the liver during necropsy. Tissue samples of a maximum size of 1 mm^3^ were taken and prefixed in phosphate-buffered (pH 7.2) 2.5% glutaraldehyde for 6 h. The samples were washed with the same buffer. Post-fixation was performed with phosphate-buffered 1% osmium tetroxide for 2 h. After dehydration with ethanol, embedding in epoxy resin, polymerizing in an oven with 45 °C for 12 h and 60 °C for 36 h, thin-sectioning with an ultramicrotome and post-staining with uranyl acetate and lead citrate, the thin sections were observed using a transmission electron microscope (JEM-1200EX, JEOL, Tokyo, Japan).

### 4.10. Assessment of Mitochondrial Membrane Potential

The electrical transmembrane potential of mitochondria was monitored with a mitochondrial membrane potential assay kit with JC-1 (Jiancheng Bioengineering Institute, Nanjing, China) using a fluorescence microscope (Ti-U, Nikon, Tokyo, Japan) and a fluorescence spectrophotometer (LS-55, PerkinElmer, San Francisco, CA, USA).

### 4.11. Assessment of CytC Release

The concentration of CytC in the endochylema was determined with a commercial ELISA Kit (CUSABIO, Wuhan, China) using multiscan spectrophotometry to detect the absorbance at 540 nm and the concentration was calculated according to the standard curve.

### 4.12. Statistical Analysis

All values are expressed as mean ± standard error of the mean (SEM). Data were analyzed using a one-way analysis of variance (ANOVA) with Dunnett’s multiple comparisons, with *p* values < 0.05 considered to be statistically significant.

## 5. Conclusions

In summary, our data suggest that aqueous extract of EF can induce oxidative damage in rat mitochondria, result in mitochondria swelling, vacuolation, MPT pore opening and mitochondrial potential decreases, demonstrating that EF induces MPT, finally resulting in ATP depletion and CytC release, triggering cell death signaling pathways, which are all partial hepatotoxicity mechanisms of EF.

## References

[1] National Pharmacopoeia Committee (2010). Pharmacopoeia of People’s Republic of China, Part 1.

[2] Jia S., Hu C. (2010). Pharmacological effects of rutaecarpine as a cardiovascular protective agent. Molecules.

[3] Chien C.C., Wu M.S., Shen S.C., Ko C.H., Chen C.H., Yang L.L., Chen Y.C. (2014). Activation of JNK contributes to evodiamine-induced apoptosis and G2/M arrest in human colorectal carcinoma cells: A structure-activity study of evodiamine. PLoS One.

[4] Ogasawara M., Matsubara T., Suzuki H. (2001). Screening of natural compounds for inhibitory activity on colon cancer cell migration. Biol. Pharm. Bull..

[5] Xu M.L., Li G., Moon D.C., Lee C.S., Woo M.H., Lee E.S., Jahng Y., Chang H.W., Lee S.H., Son J.K. (2006). Cytotoxicity and DNA topoisomerase inhibitory activity of constituents isolated from the fruits of *Evodia*
*officinalis*. Arch. Pharm. Res..

[6] Xu Y., Liu Q., Xu Y., Liu C., Wang X., He X., Zhu N., Liu J., Wu Y., Li Y. (2014). Rutaecarpine suppresses atherosclerosis in ApoE^−/−^ mice through upregulating ABCA1 and SR-BI within RCT. J. Lipid Res..

[7] Sheu J.R. (1999). Pharmacological effects of rutaecarpine, an alkaloid isolated from *Evodia*
*rutaecarpa*. Cardiovasc. Drug Rev..

[8] Tan M.X., Liu Y.C., Luo X.J., Li D.Q. (2012). Studies on the antioxidant activities of total alkaloids from the fruits of *Evodia*
*rutaecarpa* (Juss.) Benth. Adv. Mater. Res..

[9] Cai X.Y., Meng N., Yang B. (2006). Analysis of one poisoning case caused by excessive *Evodiae fructus*. Beijing Tradit. Chin. Med..

[10] Cohen S.M., Heywood E., Pillai A., Ahn J. (2012). Hepatotoxicity associated with the use of White Flood, a nutritional supplement. Pract. Gastroenterol..

[11] Teschke R. (2014). Traditional Chinese Medicine induced liver injury. J. Clin. Translat. Hepatol..

[12] Teschke R., Wolff A., Frenzel C., Schulze J. (2014). Letter: Herbal hepatotoxicity—An update on traditional Chinese medicine preparations; authors’s reply. Aliment. Pharmacol. Ther..

[13] Yang X.W. (2008). Toxicological assessment on safety of water and 70% ethanolic extracts of nearly ripe fruit of *Evodia*
*rutaecarp*a. Zhongguo Zhong Yao Za Zhi.

[14] Zhou Q., Zhang Q., Jin R.M. (2011). Time-effect and dose-effect of *Evodia*
*rutaecarpa* on hepatotoxicity in mice. Chin. J. Exp. Tradit. Med. Formulae.

[15] Huang W., Sun R. (2013). Study on chronic toxicity of water extraction components from *Evodia**fructus* in Rats. Chin. J. Exp. Tradit. Med. Formulae.

[16] Huang W., Li X., Sun R. (2012). “Dose-time-toxicity” relationship study on hepatotoxicity caused by multiple dose water extraction components of Evodiae Fructus to mice. Zhongguo Zhong Yao Za Zhi.

[17] Kim D., Lee Y.H., Park S.H., Lee M.J., Kim M.J., Jang H.S., Lee J.M., Lee H.Y., Han B.S., Son W.C. (2014). Subchronic oral toxicity of evodia fruit powder in rats. J. Ethnopharmacol..

[18] Li L. (2007). Research progress on mechanism of drug-induced hepatotoxicity. Fudan Univ. J. Med. Sci..

[19] Deng X., Luyendyk J.P., Ganey P.E., Roth R.A. (2009). Inflammatory stress and idiosyncratic hepatotoxicity: Hints from Animal Models. Pharmacol. Rev..

[20] Jaeschke H., Gores G.J., Cederbaum A.I., Hinson J.A., Pessayre D., Lemasters J.J. (2002). Mechanisms of hepatotoxicity. Toxicol. Sci..

[21] Jaeschke H., Williams C.D., Ramachandran A., Bajt M.L. (2012). Acetaminophen hepatotoxicity and repair: the role of sterile inflammation and innate immunity. Liver Int..

[22] Masubuchi Y., Kano S., Horie T. (2006). Mitochondrial permeability transition as a potential determinant of hepatotoxicity of antidiabetic thialozidinediones. Toxicology.

[23] Masubuchi Y., Suda C., Horie T. (2005). Involvement of mitochondrial permeability transition in acetaminophen-induced liver injury in mice. J. Hepatol..

[24] Labbe G., Pessayre D., Fromenty B. (2008). Drug-induced liver injury through mitochondrial dysfunction: Mechanisms and detection during preclinical safety studies. Fundam. Clin. Pharmacol..

[25] Pessayre D., Mansouri A., Berson A., Fromenty B. (2010). Mitochondrial involvement in drug-induced liver injury. Handb. Exp. Pharmacol..

[26] Teschke R., Wolff A., Frenzel C., Schulze J. (2014). Review article: Herbal hepatotoxicity—An update on traditional Chinese medicine preparations. Aliment. Pharmacol. Ther..

[27] Lee W.M. (2003). Drug-induced hepatotoxicity. N. Engl. J. Med..

[28] Jaeschke H., McGill M.R., Ramachandran A. (2012). Oxidant stress, mitochondria, and cell death mechanisms in drug-induced liver injury: Lessons learned from acetaminophen hepatotoxicity. Drug Metab. Rev..

[29] Zhang T., Qu S., Shi Q., He D., Jin X. (2014). Evodiamine induces apoptosis and enhances TRAIL-induced apoptosis in human bladder cancer cells through mTOR/S6K1-mediated downregulation of Mcl-1. Int. J. Mol. Sci..

[30] Zhang Q., Zhou Q., Jin R.M., Yao G.T., Chen X.M. (2011). Preliminary study on hepatotoxicity and nephrotoxicity induced by rutaecarpine. Chin. J. Exp. Tradit. Med. Formulae.

[31] Zhou Q., Jin R.M., Yao G.T. (2013). Preliminary study on nephrocytes toxicity induced by four traditional Chinese medicine monomers in evodia rutaecarpa. Chin. J. Pharmacovigil..

[32] Negre-Salvayre A., Auge N., Ayala V., Basaga H., Boada J., Brenke R., Chapple S., Cohen G., Feher J., Grune T. (2010). Pathological aspects of lipid peroxidation. Free Radic. Res..

[33] Watson W.H., Yang X., Choi Y.E., Jones D.P., Kehrer J.P. (2004). Thioredoxin and its role in toxicology. Toxicol. Sci..

[34] Kasznicki J., Kosmalski M., Sliwinska A., Mrowicka M., Stanczyk M., Majsterek I., Drzewoski J. (2012). Evaluation of oxidative stress markers in pathogenesis of diabetic neuropathy. Mol. Biol. Rep..

[35] Ramachandran A., Lebofsky M., Baines C. P., Lemasters J.J., Jaeschke H. (2011). Cyclophilin D deficiency protects against acetaminophen-induced oxidant stress and liver injury. Free Radic. Res..

[36] Zamzami N., Hirsch T., Dallaporta B., Petit P.X., Kroemer G. (1997). Mitochondrial implication in accidental and programmed cell death: Apoptosis and necrosis. J. Bioenerg. Biomembr..

[37] Trost L.C., Lemasters J.J. (1996). The mitochondrial permeability transition: a new pathophysiological mechanism for Reye’s syndrome and toxic liver injury. J. Pharmacol. Exp. Ther..

[38] Masubuchi Y., Nakayama S., Horie T. (2002). Role of mitochondrial permeability transition in diclofenac-induced hepatocyte injury in rats. Hepatology.

[39] Haasio K., Lounatmaa K., Sukura A. (2002). Entacapone does not induce conformational changes in liver mitochondria or skeletal muscle *in vivo*. Exp. Toxic. Pathol..

[40] Haasio K., Koponen A., Penttilä K.E., Nissinen E. (2002). Effects of entacapone and tolcapone on mitochondrial membrane potential. Eur. J. Pharmacol..

[41] Green D.R., Reed J.C. (1998). Mitochondria and apoptosis. Science.

[42] Bae M.A., Song B.J. (2003). Critical role of c-Jun N-terminal protein kinase activation in troglitazone-induced apoptosis of human HepG2 hepatoma cells. Mol. Pharmacol..

[43] Gunawan B.K., Liu Z.X., Han D., Hanawa N., Gaarde W.A., Kaplowitz N. (2006). c-Jun N-terminal kinase plays a major role in murine acetaminophen hepatotoxicity. Gastroenterology.

